# Shift in Conformational Equilibrium Underlies the Oscillatory Phosphoryl Transfer Reaction in the Circadian Clock

**DOI:** 10.3390/life11101058

**Published:** 2021-10-08

**Authors:** Pyonghwa Kim, Neha Thati, Shreya Peshori, Hye-In Jang, Yong-Ick Kim

**Affiliations:** 1 Department of Chemistry and Environmental Science, New Jersey Institute of Technology, Newark, NJ 07102, USA; pk479@njit.edu; 2 Department of Biological Sciences, New Jersey Institute of Technology, Newark, NJ 07102, USA; nrt9@njit.edu (N.T.); sp2578@njit.edu (S.P.); 3 School of Cosmetic Science and Beauty Biotechnology, Semyung University, Jecheon 27136, Korea; 4 Institute for Brain and Neuroscience Research, New Jersey Institute of Technology, Newark, NJ 07102, USA

**Keywords:** kinase, phosphatase, KaiB, phosphorylation, dephosphorylation, circadian clock

## Abstract

Oscillatory phosphorylation/dephosphorylation can be commonly found in a biological system as a means of signal transduction though its pivotal presence in the workings of circadian clocks has drawn significant interest: for example in a significant portion of the physiology of *Synechococcus elongatus* PCC 7942. The biological oscillatory reaction in the cyanobacterial circadian clock can be visualized through its reconstitution in a test tube by mixing three proteins—KaiA, KaiB and KaiC—with adenosine triphosphate and magnesium ions. Surprisingly, the oscillatory phosphorylation/dephosphorylation of the hexameric KaiC takes place spontaneously and almost indefinitely in a test tube as long as ATP is present. This autonomous post-translational modification is tightly regulated by the conformational change of the C-terminal peptide of KaiC called the “A-loop” between the exposed and the buried states, a process induced by the time-course binding events of KaiA and KaiB to KaiC. There are three putative hydrogen-bond forming residues of the A-loop that are important for stabilizing its buried conformation. Substituting the residues with alanine enabled us to observe KaiB’s role in dephosphorylating hyperphosphorylated KaiC, independent of KaiA’s effect. We found a novel role of KaiB that its binding to KaiC induces the A-loop toward its buried conformation, which in turn activates the autodephosphorylation of KaiC. In addition to its traditional role of sequestering KaiA, KaiB’s binding contributes to the robustness of cyclic KaiC phosphorylation by inhibiting it during the dephosphorylation phase, effectively shifting the equilibrium toward the correct phase of the clock.

## 1. Introduction

Amidst growing global concerns over greenhouse gas emissions and a potential energy crisis, a growing number of studies are focusing on possible ways to harvest electrons [1,2] and chemicals [3,4] from cyanobacteria through bioengineering. For billions of years these unheralded bacteria have been using solar energy to capture carbon dioxide and split water, contributing to the oxygenic atmosphere that we breathe today [5]. Among them is *Synechococcus elongatus* PCC 7942, of which a significant portion of its physiology is regulated by a circadian clock; that is, the expression of light-harvesting proteins peaks just before sunrise and that of proteins needed to break down stored glycogen right before sunset [6,7,8].

Equipped with the biological intricacy that provides a fitness advantage in a rhythmically oscillating environment [9], cyanobacteria have a central oscillator that is composed of three proteins—KaiA, KaiB, and KaiC—which generate cyclic phosphorylation and dephosphorylation of KaiC with ~24 h periodicity [10]. KaiC displays both autokinase and autophosphatase activities regulated by the conformation of its C-terminal peptide called the “A-loop” [11,12]. The two conformations (exposed and buried) of the A-loop equilibrate dynamically (Figure S1), triggering yet another structural variation in the active sites of KaiC [13,14]. We have previously suggested that this latter arrangement modulates the binding affinity of magnesium ions (Mg^2+^), which in turn regulates the autokinase and autophosphatase activities in KaiC [14,15]. When the A-loop is exposed, the Mg^2+^ binding affinity decreases and the autokinase activity is activated [13]. On the other hand, the autophosphatase is activated when the A-loop takes the buried conformation and increases the affinity for binding Mg^2+^ in the active sites [14]. KaiA shifts the dynamic equilibrium of the A-loop toward the exposed state by binding to its unraveled form. This conformational locking induces dissociation of Mg^2+^ from the active sites and initiates KaiC autophosphorylation. On the other hand, KaiB induces dephosphorylation by sequestrating KaiA from the A-loop, shifting the dynamic equilibrium toward the buried conformation, supposedly due to a decrease in the abundance of free KaiA, which can activate phosphorylation by binding the A-loop [13,16].

Surprisingly, this unusual biological oscillatory system can be reconstituted in a test tube by mixing the three Kai proteins with adenosine triphosphate (ATP) [17,18,19]. By examining the simple oscillatory reaction in vitro, the role of KaiA has been elucidated comparatively well but so far that of KaiB is known to be limited to sequestering KaiA. To study the function of KaiB on the oscillatory phosphorylation and dephosphorylation of KaiC, studies have been performed with the three-component KaiABC system although its complexity can hinder attempts to isolate the role of an individual component. In this paper, we showed evidence of the self-regulatory reaction mechanism by applying single mutations on the KaiC protein. We applied three alanine mutations individually on the A-loop’s hydrogen bonding (H-bonding) residues that are known to stabilize its buried conformation to shift the equilibrium toward the exposed conformation so that each mutant KaiC displayed a constitutively phosphorylated state even without KaiA. Using these KaiC mutants to reconstitute the two-component KaiBC systems in vitro enabled us to investigate the role of KaiB more thoroughly in the circadian oscillator.

## 2. Materials and Methods

### Phosphorylation Assay of the Circadian Clock In Vitro

Cloning, purifications, and phosphorylation assays were performed as described previously with minor modifications [18,19]. Basically, the KaiC mutant plasmids for protein expression are generated by using the site-directed mutagenesis with the pET41a(+)-KaiC plasmid as a template. Disodium ATP was used for all reaction buffers including protein purification buffers to avoid adding Mg^2+^ from another reagent. The phosphorylation states of KaiC (p-KaiC, %) were measured by the gel densitometry using ImageJ software (NIH) [20]. Further details are provided in SI Text.

## 3. Results

### 3.1. Breaking H-bonds between the A-loops Induces the KaiC Phosphorylation

**Figure 1:**
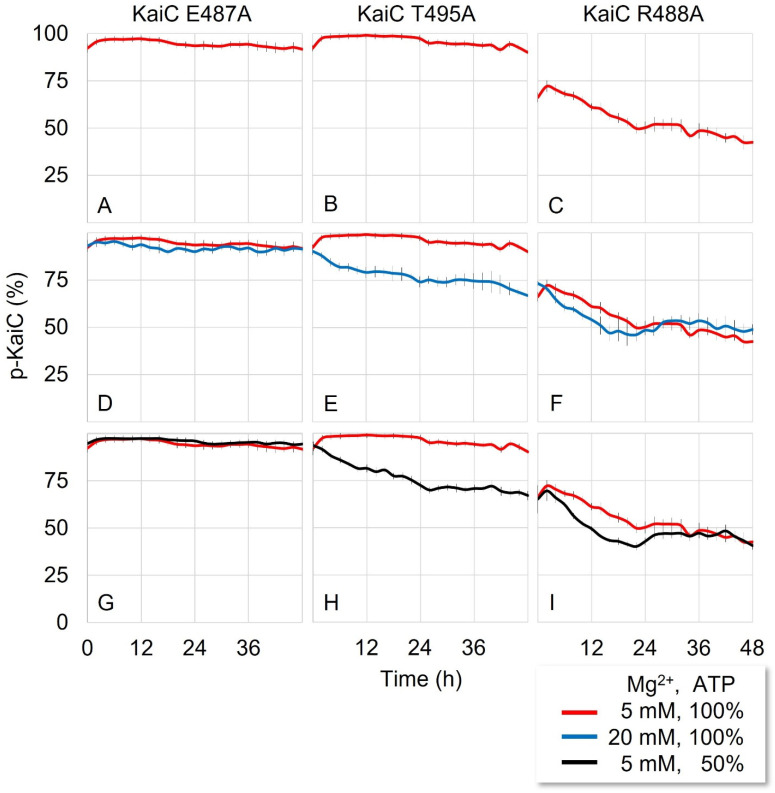
**Breaking the H-bond network on the A-loop induces the phosphorylation of KaiC mutants.** (**A**–**I**) All reactions were performed in the same buffer condition (150 mM NaCl, 20 mM Tris-HCl, 5 mM MgCl_2_, 0.5 mM EDTA, 1 mM ATP, pH = 8.0). If the concentration was not specified. KaiC (3.4 µM) was the only protein in the reaction mixture. Graphs are the average of the two or three replicates. Vertical bars are the standard errors of the mean (SEM) on the data points. The raw data of the gels are given in Figure S4.

The autokinase activity of KaiC is regulated by the conformation of its A-loop [13]. In the hexameric KaiC, the buried conformation of the A-loop is stabilized by forming inter- and intra-subunit H-bonds, and breaking these interactions enhances the chance to stay exposed [21,22]. This dynamic conformational equilibrium affects the Mg^2+^ binding in the active sites, resulting in the regulation of the autophosphorylation or autodephosphorylation of KaiC [14]. To observe the H-bonding effect on the KaiC phosphorylation, we analyzed the crystal structure of KaiC (PDBID:1TF7) [23] and selected three putative H-bond forming residues (E487, R488, and T495) on the A-loop for mutation studies (Figure S2). By replacing the original residues to alanine, the H-bonds may be broken in the hexameric KaiC. With the three KaiC mutants—KaiC-E487A, KaiC-R488A and KaiC-T495A (hereafter KaiC^E487A^, KaiC^R488A^, and KaiC^T495A^), respectively—reaction mixtures were reconstituted by following the established in vitro protocol to monitor the phosphorylation states [18]. In the absence of KaiA and KaiB, KaiC–wild type (WT) is known to dephosphorylate [13]. For our KaiC mutants, KaiC^E487A^ (Figure 1A) and KaiC^T495A^ (Figure 1B) started phosphorylation at the beginning of the reaction and remained hyperphosphorylated (>95% phosphorylation) for two days. However, KaiC^R488A^ (Figure 1C) underwent phosphorylation for the first 2 h (although the change is not significant) and slowly dephosphorylated for 12 h until it reached ~50% phosphorylation.

In general, to stimulate KaiC phosphorylation in vitro, KaiA had to be added to the regular reaction mixture [17,18] or the Mg^2+^ concentration was lowered in the absence of ethylenediaminetetraacetic acid (EDTA) [14]. By breaking the H-bonds on the A-loop of KaiC, the kinase activity was activated without adding KaiA. From this result, we propose that breaking the H-bonds shifts the dynamic equilibrium of A-loop conformation toward the exposed conformation for KaiC^E487A^ and KaiC^T495A^, which activates the autokinase activity and suppress the autophosphatase activity normally present in wild-type KaiC (Figure S3). Based on the phosphorylation profile, we predicted that E487 and T495 would form strong H-bonds while R488 would make comparatively weak H-bonds in the A-loop.

### 3.2. The KaiC Dephosphorylation Was Enhanced in High Mg^2+^ Concentration

KaiC also had autophosphatase, in addition to autokinase, activity. We previously reported that Mg^2+^ concentration is a crucial factor for regulating autokinase and autophosphatase activity [14]. In the low or high concentration of Mg^2+^, KaiC phosphorylates or dephosphorylates respectively even without KaiA and KaiB. However, the excess amount of Mg^2+^ did not change the phosphorylation state of KaiC-WT (Figure S5). To observe the Mg^2+^ effect on the phosphorylation state of KaiC^E487A^, KaiC^R488A^, and KaiC^T495A^ we added 20 mM Mg^2+^ (four times higher than the regular concentration) to the reaction mixture. Because its high concentration induced the dephosphorylation of KaiC [14], we expected that Mg^2+^ would attenuate the kinase activity of the hyperphosphorylated KaiC mutants (KaiC^E487A^ and KaiC^T495A^) and lead to some degree of dephosphorylation. The effect was not observed for KaiC^E487A^ with 20 mM Mg^2+^ (Figure 1D). However, KaiC^T495A^ showed dephosphorylation in the beginning and for 48 h remained at ~75% phosphorylation (Figure 1E), which is ~20% lower than that of the regular condition (5 mM Mg^2+^). In the case of KaiC^R488A^, dephosphorylation was slightly enhanced by higher Mg^2+^ at the beginning but stopped at the same level (~50% phosphorylation) after 12 h (Figure 1F), in a manner similar to that of the regular condition (5 mM Mg^2+^).

In sum, higher Mg^2+^ concentration enhanced the dephosphorylation of KaiC^T495A^ and KaiC^R488A^ but its effect on KaiC^E487A^ was miniscule. We previously suggested that the dephosphorylation was induced when Mg^2+^ was bound in the active site and its affinity was increased or decreased with the buried or exposed conformation of A-loop, respectively [14]. Therefore, the dynamic equilibrium of A-loop conformations could be predicted based on phosphorylation levels of the KaiC mutants. For KaiC^E487A^, the equilibrium could not be shifted toward the buried conformation even at high Mg^2+^ concentration (Figure S6) because severing the seemingly strong glutamate H-bond exposed the A-loop by making it irreversibly flexible and unperturbed by the presence of Mg^2+^ [21]. On the other hand, for KaiC^T495A^, the A-loop’s dynamic equilibrium partially shifted toward the buried conformation, with which more abundant Mg^2+^ had a better chance of binding KaiC and activating dephosphorylation, following the Le Chatelier’s principle (Figure S6). The dynamic equilibrium seemed balanced in KaiC^R488A^ since its phosphorylation level stagnated at ~50%.

### 3.3. Decreasing Adenosine Triphosphate (ATP) Ratio Enhanced the KaiC Dephosphorylation

Decreasing ATP or increasing the adenosine diphosphate (ADP) ratio in the nucleotides pool can also induce the dephosphorylation of KaiC. A temporary drop in the ATP ratio to 50% during the phosphorylation phase is known to induce dephosphorylation [24]. However, increasing ADP does not affect the dephosphorylation of KaiC-WT [24]. Unlike Mg^2+^, ATP and ADP are used not as regulators but as reactants for phosphorylation and dephosphorylation reactions [14,15,25]. We investigated the ATP ratio effect on the KaiC phosphorylation with KaiC^E487A^, KaiC^R488A^, and KaiC^T495A^. The KaiC mutants were dephosphorylated with increasing ADP, in a similar manner as that of increasing Mg^2+^ concentration (Figure 1G–I). Because ATP and ADP are reactants in the phosphorylation and dephosphorylation reaction of KaiC, adding ADP could not directly shift the dynamic equilibrium of A-loop conformation (Figure S7). However, phosphorylation was inhibited when ADP was bound to the CII domain of KaiC, even when the A-loop’s conformation was exposed; conversely if ADP bound to KaiC when the A-loop was buried, dephosphorylation would proceed [15].

Based on the results of the mutation studies, we can predict how much of each A-loop conformation is favored in the dynamic equilibrium for the KaiC mutants. The A-loop of KaiC^E487A^ seemed to stay constantly exposed since the presence of ADP was not able to induce dephosphorylation. However, KaiC^E495A^ and KaiC^R488A^ had the dynamic equilibrium slightly favored toward the buried state, although the former’s case was less so. Therefore, the H-bond of E487 would be the highest determinant, and that of R488 the lowest, for stabilizing the buried conformation of the A-loop.

### 3.4. KaiB Attenuates the Phosphorylation Activated by Breaking the H-bonds in A-loop

**Figure 2:**
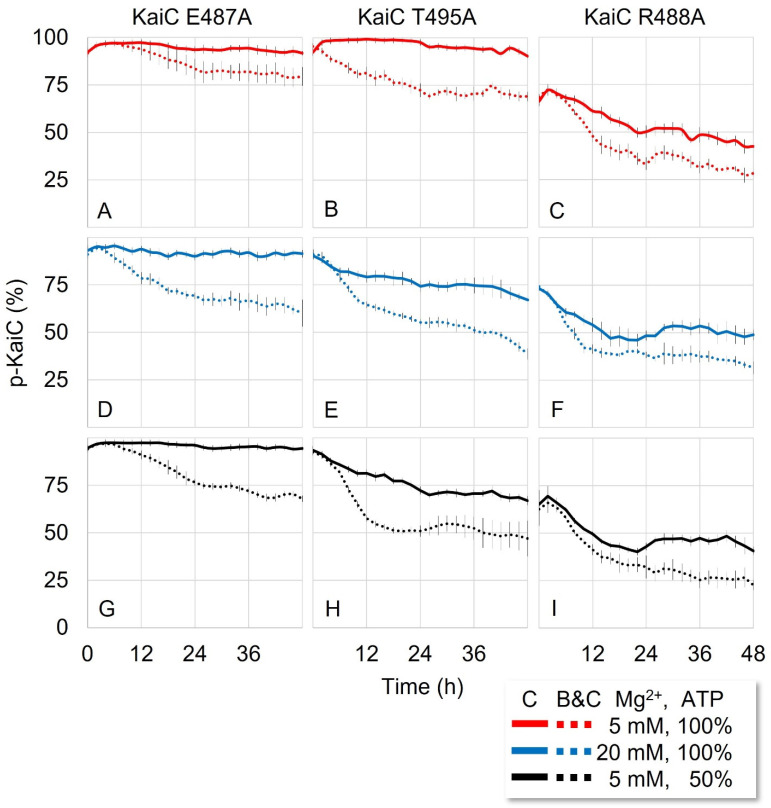
**KaiB binding locks the buried conformation of A-loop inducing the dephosphorylation of KaiC mutants.** (**A**–**I**) All are the same as Figure 1 except that KaiB (3.4 µM) was added to the reaction mixture. The raw data of the gels are in Figure S4.

Traditionally, KaiB is known to induce the dephosphorylation of KaiC indirectly by sequestering KaiA from the A-loop [16,21,26]. Because no direct effect of KaiB on the KaiC phosphorylation was reported [13], we used the KaiC mutants that remained constitutively hyperphosphorylated in the absence of KaiA to examine the effect. When KaiB was added to the reaction mixtures containing only the KaiC mutants, all of them showed dephosphorylation to a certain degree. Interestingly, KaiC^E487A^, which was unable to be dephosphorylated with other treatments, showed dephosphorylation in the presence of KaiB (Figure 2A). KaiC^T495A^ displayed the largest effect among the mutants (~20%) although the magnitude was smaller than that of the KaiC wild type (Figure 2B). KaiC^R488A^ also showed ~10% more dephosphorylation in the presence of KaiB (Figure 2C). Because the phosphorylation and dephosphorylation were determined by the A-loop conformation, adding KaiB must have induced dephosphorylation on the KaiC mutants by shifting the dynamic equilibrium toward the buried conformation (Figure S2).

We also examined the Mg^2+^ effect in the presence of KaiB. Adding Mg^2+^ enhanced the dephosphorylation in all KaiC mutants in the presence of KaiB (Figure 2D–F). High Mg^2+^ concentration shifted the dynamic equilibrium more toward the buried conformation, an effect additive to the KaiB effect. It is also worth noting that KaiC^E487A^ which seemed impossible to dephosphorylate underwent dephosphorylation and became responsive to ADP and Mg^2+^ only when KaiB was present (2A, 2D, 2G). Increasing the ADP ratio also induced further dephosphorylation of KaiC^T495A^ and KaiC^R488A^ in the presence of KaiB (Figure 2G–I). Therefore, KaiB binding shifted the dynamic equilibrium toward the buried conformation even on KaiC^E487A^, which was unable to shift the equilibrium with any other treatments.

Based on this observation, KaiB directly induced the dephosphorylation of KaiC through at least one additional mechanism other than the sequestration of KaiA, which is an indirect effect. These two functional properties of KaiB may have brought about a synergetic effect on the dephosphorylation of KaiC at the onset of the dephosphorylation phase, contributing to making the oscillation more robust.

### 3.5. CII Flexibility Governs the Damped Oscillatory Phosphorylation in KaiC^E488A^

**Figure 3:**
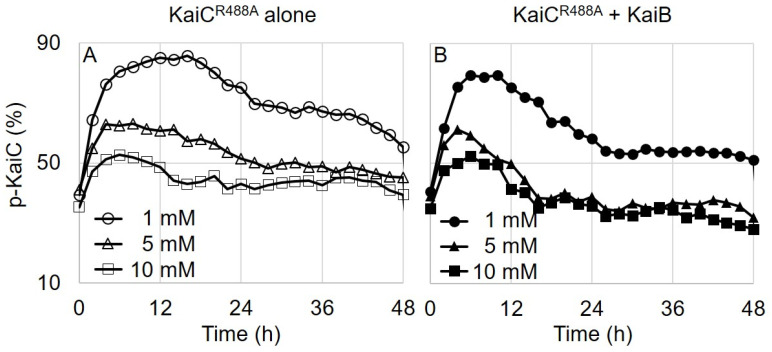
**The spontaneous phosphorylation and dephosphorylation ofKaiC^R488A^.** The phosphorylation state of KaiC^R488A^ was measured without KaiA and KaiB every 2 h for 2 days. The Mg^2+^ concentration of each reaction is labeled on the graph (**A**). The phosphorylation state of KaiC^R488A^ in the presence of KaiB (**B**). Another replicate shows similar patterns (Figure S10).

The phosphorylation profile of KaiC^R488A^ was the most intriguing because of its balance between spontaneous phosphorylation and dephosphorylation: remaining phosphorylated for the first 2 h and reaching a steady-state, mildly elevated (~50%) level (Figure 1C). It seems to have the characteristic of a damped oscillator for the KaiC^E488A^ alone reaction. We previously reported that changing the Mg^2+^ concentration in the reaction mixture modulated the phosphorylation and dephosphorylation of KaiC [14]. It seemed that if we decreased the Mg^2+^ concentration, we would have observed a higher (or longer) phosphorylation state for the KaiC^R488A^ alone reaction. To test the Mg^2+^ effect on the phosphorylation of KaiC^R488A^, we monitored the phosphorylation states in different Mg^2+^ concentrations in the absence or presence of KaiB. The unusual balance of KaiC^R488A^ between the two antagonistic reactions gave it a unique phosphorylation pattern. At low Mg^2+^ concentration (1 mM), it distinctively showed kinase activity for 12 h and underwent dephosphorylation afterwards in the KaiC^R488A^-alone reaction mixture (Figure 3A). When KaiB was present in the reaction mixture, dephosphorylation started earlier than when it was absent (Figure 3B). This abnormal behavior could have come from the flexibility change on the CII domain of KaiC throughout the phosphorylation state [27]. As hypophosphorylated KaiC became fully phosphorylated, the CII domain rigidified, and the A-loop’s dynamic equilibrium shifted from the exposed to the buried conformation (Figure S8). Therefore, KaiC^R488A^ autophosphorylated initially (phosphorylation phase) because the A-loop was exposed in the flexible CII domain. As KaiC gradually became fully phosphorylated, the CII domain rigidified and the A-loop preferred the buried conformation, eventually inducing dephosphorylation (Figure S9).

The binary KaiC–KaiB complexation seemed to affect the KaiC mutants in an irreversible manner unlike that of ADP or Mg^2+^ as both KaiC^E487A^ and KaiC^R488A^ consequently showed a higher degree of dephosphorylation when KaiB was present. This bolstered the notion that KaiC–KaiB binding locked the rigid CII domain and eventually shifted the conformational equilibrium of A-loop toward its buried state. KaiB’s binding KaiC and inducing the A-loop burial ensured that dephosphorylation would take place at the correct phase of the clock and the biological timekeeping would run clockwise.

## 4. Discussion

The cyanobacterial circadian oscillator showed oscillatory phosphorylation/dephosphorylation within ~24 h. To induce autophosphorylation or autodephosphorylation of KaiC, the A-loop conformation’s stabilization in the exposed or buried conformation led to phosphorylation or dephosphorylation, respectively. Various interactions among the Kai proteins sequentially regulated the A-loop’s conformation to generate an oscillatory KaiC phosphorylation and dephosphorylation. When KaiC was unphosphorylated, KaiA binding to the A-loop shifted the dynamic equilibrium toward the exposed conformation, which activated the KaiC phosphorylation. By breaking the inter- and intrasubunit H-bonds on the A-loop, KaiC was phosphorylated without KaiA. When KaiC was fully phosphorylated, KaiB bound to KaiC and sequestered KaiA from the A-loop. The dissociation of KaiA shifted the dynamic equilibrium toward the buried conformation, which induced the dephosphorylation of KaiC.

However, a significant amount of free KaiA protein was still present in the reaction mixture when KaiC was ready to start the dephosphorylation phase [26,28]. A decrease in free KaiA due to sequestration by KaiB would not necessarily have reversed the kinase reaction; rather, it would have just slowed it down [24,29]. In other words, with a lower amount of active KaiA in the mixture, the level of phosphorylated KaiC increased slowly, but its reduction did not trigger dephosphorylation. Therefore, the sequestration of KaiA alone was not enough to explain fully the transition and the oscillator’s clockwise sequential phosphorylation and dephosphorylation [30,31,32]. Here, we found the additional functionality of KaiB that plays a key role in the transition between the KaiC phosphorylation and dephosphorylation phases.

**Figure 4:**
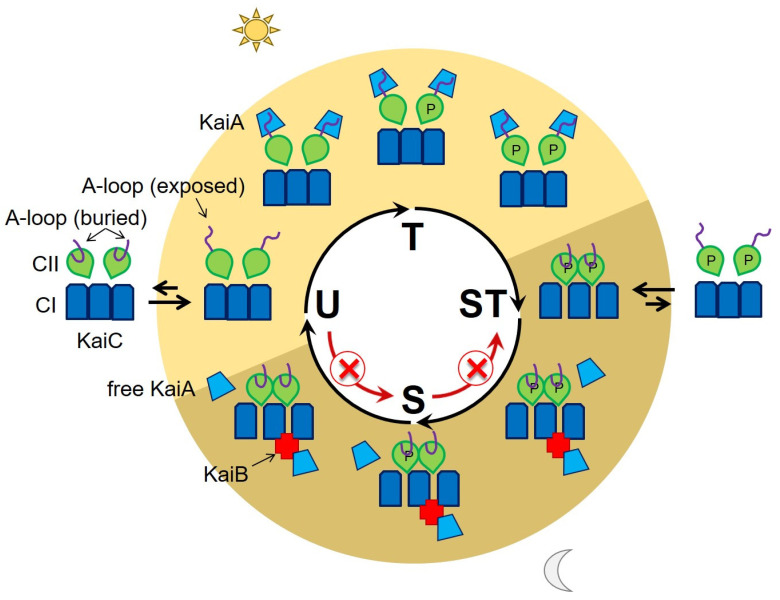
**The mechanistic model of the circadian oscillator in cyanobacteria.** To initiate dephosphorylation, the A-loop conformation is buried in the rigid CII domain (ST). KaiB binding to CI domain locks the buried conformation to keep the dephosphorylation of KaiC. The major phosphorylation states of KaiC are written on the white circle. The protein interactions corresponding to the phosphorylation states are shown on the colored (light and dark yellow) circle. To simplify, only 3 subunits of the CI domain and 2 subunits of the CII domain are drawn.

After introducing KaiB’s novel function, we proposed a detailed mechanism of the circadian oscillation at the mechanistic level (Figure 4). When KaiC was in an unphosphorylated state (U), the CII domain remained flexible and the dynamic equilibrium of the A-loop conformation shifted toward the exposed [27]. KaiA bound the exposed A-loop and KaiC phosphorylated itself until it was fully phosphorylated [12,13] (U→T→ST). When the KaiC phosphorylation peaked (ST), its CII domain became rigid and was stacked on the CI domain [32]. This conformational change shifted the A-loop’s dynamic equilibrium toward the buried conformation and KaiC started to dephosphorylate. KaiB bound to the CI domain of KaiC and locked the conformation continuing dephosphorylation (ST→S→U). Although a significant amount of free KaiA was present in the oscillatory reaction mixture at this point, the locked conformation did not allow KaiA to bind the A-loop. When KaiC was unphosphorylated (U), KaiB dissociation allowed the CII domain to be conformationally flexible, and KaiC restarted the phosphorylation cycle (U→T→ST).

To mimic hypophosphorylated (U) and hyperphosphorylated (ST) KaiC, alanine (KaiC^AA^) and glutamate (KaiC^EE^) substitutions were applied respectively to both phosphorylation sites S431 and T432 [33,34]. Surprisingly, previously reported fluorescence anisotropic data suggested that KaiC^EE^ had a greater affinity for KaiA than KaiC^AA^, in the absence of KaiB [26]. It means that the free KaiA proteins could bind to the A-loop of ST-KaiC, inhibit the dephosphorylation (ST→S) and stay on the hyperphosphorylated KaiC. Then, KaiB’s role of locking the rigid conformation of the CII domain in KaiC becomes even more crucial when it came to maintaining phosphatase activity at the beginning of the dephosphorylation phase (ST→S), considering that there was still enough residual free KaiA in the mixture to offset dephosphorylation. KaiA’s differential affinity to KaiC had previously been reported, and it was argued that it depended on the phosphorylation state of KaiC; this ensured that the oscillator ran clockwise during the phosphorylating phase [35,36]. However, the use of phosphomimetics only provided a partial understanding of the dynamic behavior of the A-loop, KaiC, and its kinase and phosphatase activities, leaving it difficult to pinpoint the cause and effect of the post-translational modification. Our finding here adds another factor that contributes to the clockwise “ticking”: KaiB’s binding KaiC allosterically induces the conformational burial of the A-loop, shifting the equilibrium towards dephosphorylation.

Among many targets, the hyperphosphorylated KaiC–KaiB complex in *S. elongatus* suppressed *kaiBC* transcription. The resultant reduction in KaiC and KaiB concentrations created a transcription–translation feedback loop (TTFL), ensuring that their production peaks only when unphosphorylated KaiC dominates at dawn. The hallmark of KaiABC-based post-translational oscillator (PTO) is that it can withstand significant cellular noise coming from various sources, including variations in the abundance of clock components [37]. Overexpression of KaiA induced the constitutive phosphorylation of KaiC and caused the oscillation to dampen, a pattern observed in TTFL-based KaiBC hourglass models in other organisms.

Among the central oscillator components, *kaiC* is thought to be the oldest, while *kaiA* is the youngest [38]. Thus, it is suggested that the emergence of a two-component KaiBC timekeeper—a supposed relic of the past that gave rise to today’s intricate cyanobacterial timekeeper—might have preceded that of a bona fide KaiABC circadian clock [39]. By adjusting the H-bonding network on the A-loop, we succeeded in shifting the equilibrium between the two antagonistic reactions sustaining the phosphorylation level of KaiC at various levels without the presence of KaiA, a possible feature of the KaiBC timekeeping system. In addition, KaiC^R488A^’s partial responsiveness to various cues and eventual return to its initial phosphorylation level hinted at the potential mechanism of a damped oscillator, a possible intermediate step in the development of the timekeeper [39,40]. The fact that the primary sequences of the A-loop varied between different *kaiC*-harboring species while the general motifs of *kaiC* were conserved brought our attention to the importance of the A-loop as the master regulator of the post-translational oscillator [41,42].

Recently, we proposed that a KaiC-alone hourglass can oscillate under the alternating Mg^2+^ concentrations in vitro, and that this perhaps put the emergence of Mg^2+^ homeostasis after that of the primeval timekeeper on the clock’s evolutionary timeline [14]. Since the endogenous Mg^2+^ concentration was reported to be constant in *S. elongatus* [43], the emergence of a damped oscillator as a better timekeeping system than the cue-dependent hourglass fits well in the picture [39]. After going through multiple evolutionary steps, the current self-sustained circadian oscillator may have emerged as a biologically efficient way to exploit the periodic availability of solar energy.

In addition, Mg^2+^ is a necessary element of ATPase activity on the CI domain of KaiC. The conformational change driven by the ATPase activity enabled KaiB binding and promoted dephosphorylation (Figure S11). Although we did not examine the effect of Mg^2+^ on ATPase activity in this paper, it could have affected KaiB binding by influencing ATP hydrolysis activity in the CI domain. Researching the effect of Mg^2+^ on ATPase activity will be an interesting topic for understanding how the phosphorylation states of CII are communicated to the ATPase activity on CI.

Although we stated that ADP affected phosphorylation and dephosphorylation, it is possible that ADP also remained on CI after ATP hydrolysis and facilitated KaiB binding (Figure S11). This may have been another cause of the enhanced dephosphorylation when we increased the ADP ratio. In addition to the potential effects of Mg^2+^ and ADP on CI, the effect of the fold-switched KaiB would also be interesting to examine since different KaiB binding kinetics may result from changes in the speed and magnitude of the dephosphorylation in our KaiC mutants. A more in-depth understanding of KaiB binding kinetics will move us closer to reconstituting the hourglass KaiBC timekeeper.

The unidirectional clockwise running of sequential KaiC phosphorylation and the KaiC–KaiB complexation that takes place only at the correct phase tuned the timing of the bifunctional CikA’s binding and its possible inactivation by oxidized quinone, which caused a delay in the phase response of the KaiC phosphorylation rhythm to a dark pulse application when applied in the falling phase of the clock [44]. KaiB binding‘s allosteric induction of A-loop burial also had a potential role in the entrainment process since maintaining the A-loop’s buried conformation during the falling phase of the clock was crucial not only for maintaining the KaiC–KaiB–CikA complex, but also because KaiA could not tether the A-loop in its exposed conformation and run the rhythm backward toward phosphorylation. If the A-loop became exposed at this stage, KaiA, not CikA, would have been inactivated by oxidized quinone, which would have advanced the phase instead of delaying it [43,45,46]. It is through this process that KaiB initiated the dephosphorylation phase at the correct timing of day and ensured that the CikA binding took place accordingly.

If the proliferation of cyanobacteria billions of years ago led to the Great Oxygenation that transformed Earth’s anoxic atmosphere, bioengineering cyanobacteria can be considered as one of the potential strategies to combat the environmental and energy crises. By understanding the functional and structural properties of the cyanobacterial circadian clock, we could bring the undoing the man-made carbon dioxide production closer to reality.

## Data Availability

Data are available upon request to the authors.

## Supplementary Materials

The following are available online at www.mdpi.com/2075-1729/11/10/1058/s1, Figure S1: The dynamic equilibrium of A-loop conformations. Figure S2: The putative H-bond network of A-loops within the KaiC hexamer. Figure S3: The dynamic equilibrium of KaiC mutants. Figure S4: KaiC phosphorylation with various Mg^2+^ concentrations and ATP ratio. Figure S5: The dephosphorylation of KaiC in the presence of high Mg^2+^ concentration. Figure S6: Dynamic equilibrium of KaiC mutants in the presence of high Mg^2+^ concentration. Figure S7: The dynamic equilibrium of KaiC mutants in the presence of high ADP concentration. Figure S8: The flexibility change of the CII domain of KaiC throughout each phosphorylation state. Figure S9: A model of the conformational changes during the KaiC phosphorylation and dephosphorylation. Figure S10: The spontaneous phosphorylation and dephosphorylation of KaiC^R488A^. Figure S11: Mg^2+^ and ADP involvement on the ATP hydrolysis and KaiB binding on the CI domain of KaiC.
