# Supplementary material for: Shift in Conformational Equilibrium Underlies the Oscillatory Phosphoryl Transfer Reaction in the Circadian Clock
Source: Life (Basel). 2021 Oct 8;11(10):1058. doi: 10.3390/life11101058 (PMC8538168; doi:10.3390/life11101058)
Supplement: Supplementary file 1 [file life-11-01058-s001.zip › life-1401476-supplementary.pdf]

life

MDPI

Supplementary material of Shift in Conformational Equilibrium Underlies the Oscillatory Phosphoryl Transfer Reaction in the Circadian Clock

Pyonghwa Kim [1], Neha Thati [2], Shreya Peshori [2], Hye-In Jang [3,*] and Yong-Ick Kim [1,4,*]

[1] Department of Chemistry and Environmental Science, New Jersey Institute of Technology, Newark, NJ 07102, USA; pk479@njit.edu
[2] Department of Biological Sciences, New Jersey Institute of Technology, Newark, NJ 07102, USA; nrt9@njit.edu
[3] School of Cosmetic Science and Beauty Biotechnology, Semyung University, Jecheon 27136, Korea
[4] Institute for Brain and Neuroscience Research, New Jersey Institute of Technology, Newark, NJ 07102, USA
* Correspondence: inijjang@semyung.ac.kr (H.-I.J.) and ykim@njit.edu (Y.-I.K.)

Citation: Kim, P.; Thati, N.; Peshori, S.; Jang, H.-I.; Kim, Y.-I. Shift in Conformational Equilibrium Underlies the Oscillatory Phosphoryl Transfer Reaction in the Circadian Clock. *Life* **2021**, *11*, 1058. https://doi.org/10.3390/life11101058

Academic Editor: Renate Kunert

Received: 14 September 2021
Accepted: 5 October 2021
Published: 8 October 2021

Publisher's Note: MDPI stays neutral with regard to jurisdictional claims in published maps and institutional affiliations.

Copyright: © 2021 by the authors. Licensee MDPI, Basel, Switzerland. This article is an open access article distributed under the terms and conditions of the Creative Commons Attribution (CC BY) license (http://creativecommons.org/licenses/by/4.0/).

Methods

Expression and purification of KaiC: GST-tagged KaiC mutants were made through the site directed mutagenesis PCR with pET-41a(+) KaiC-WT plasmid previously made [1]. After the plasmid was verified through DNA sequencing, it was transformed in *E. coli* (BL21DE3, Novagen) and grown in 1L of Luria Bertani (LB) media containing kanamycin. When OD_{600} of the culture reached 7.0, the temperature was lowered to 25˚C. 100 µM IPTG was added to induce overexpression of KaiC, and the culture was incubated for additional 16 hours. Cells were harvested by centrifugation at 5500 × g for 10 min. Cell pellets were resuspended in 60mL of wash buffer (50 mM Tris-HCl, 150 mM NaCl, 5 mM $MgCl_2$, 1 mM EDTA, 5 mM ATP, and 1 mM DTT at pH 7.3) and lysed by passing through a chilled French press cell at a constant rate with 16,000 psi. the lysates were centrifuged at 20,000 × g for 2 hours and filtered through a 0.45 µm filter (Thermo-Fisher). The separation of KaiC from crude cell lysate was achieved by lysing the cells and injecting cell lysate into a 5 ml prepacked GST column (GSTrap HP, GE Healthcare), where the GST-tagged KaiC selectively binds. The elution buffer contained 50 mM Tris-HCl · pH 7.3, 150 mM NaCl, 5 mM $MgCl_2$, 1 mM EDTA, 1 mM ATP, 1 mM DTT and 6 mM glutathione. After the sample elution, PreScission™ protease (GE Healthcare) was used to cleave the GST-tags from KaiC. Additionally, HiPrep 26/10 desalting columns from GE Healthcare were used for desalting steps. The final KaiC protein isolate was obtained through the use of another GST column, where digested GST-tags are separated from KaiC in solution. Bradford Reagent was combined with small samples of the final KaiC isolate to measure the protein concentration based on a predetermined standard curve. The samples were run individually through a 10% SDS-polyacrylamide gel to verify that each contains KaiC.

KaiC phosphorylation assay: After performing *in vitro* reactions and aliquoting samples at evenly spaced time intervals, the phosphorylation states of the samples were visualized through SDS-PAGE and subsequent staining using InstantBlue™ Coomassie stain (Expedeon). The *in vitro* reaction mixture was prepared by mixing 3.5 µM KaiB, 3.5 µM KaiC, 1 mM ATP, 5 mM $MgCl_2$, 150 mM NaCl, 0.5 mM EDTA, and Tris-HCl at pH 8.0 (8, 16). After aliquoting from each reaction during evenly spaced 2-hour time points over the span of 48 hours, the samples were denatured, run on a 6.5% SDS-polyacrylamide gel, and analyzed by constructing a densitometric graph of phosphorylation states.

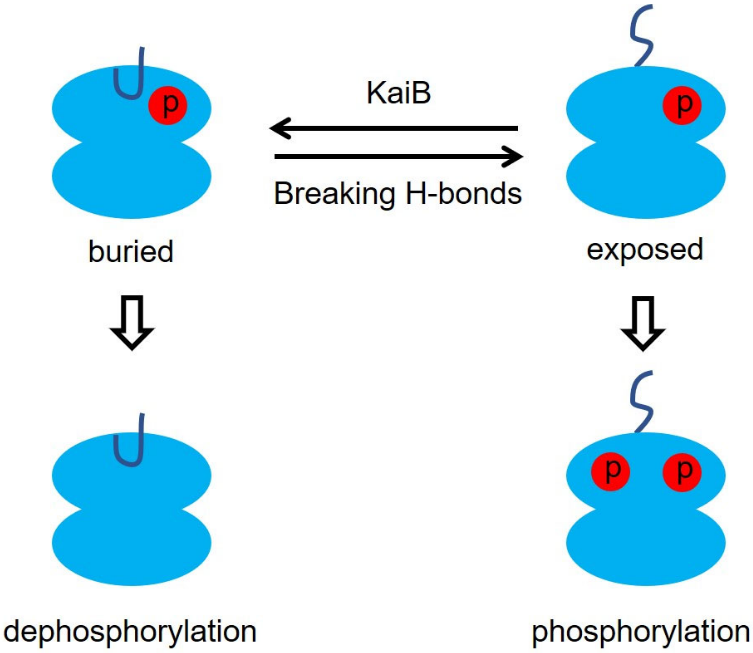

Figure S1. The dynamic equilibrium of A-loop conformations. The A-loop conformations of KaiC change spontaneously in the regular reaction condition[2]. The buried conformation and exposed conformation induce the dephosphorylation and phosphorylation of KaiC, respectively[3]. The factors shifting the equilibrium were written on the arrows. P in the red circle represents two phosphorylation sites in KaiC monomer. One A-loop and two phosphorylation were drawn on the cartoon representation of KaiC hexamer for simplicity.

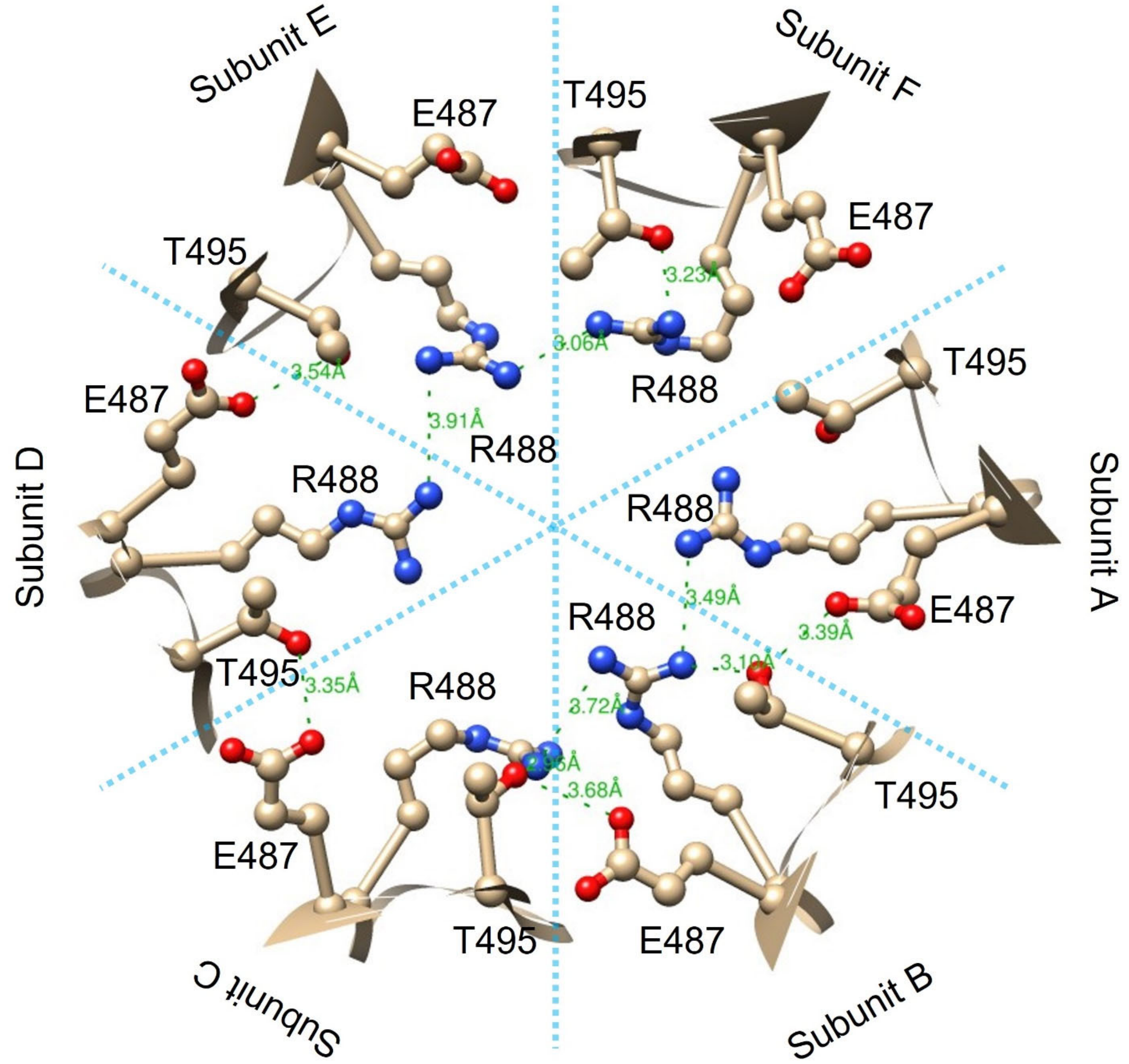

Figure S2. The putative H-bond network of A-loops within the KaiC hexamer. The KaiC X-ray crystal structure (PDBID: 1TF7) was analyzed using UCSF Chimera[4]. Each A-loop from monomer subunits is separated by blue dotted line for visibility. The atoms forming putative H-bonds are connected with green dotted lines. The distances are measured using the "Structure analysis – Distances" function in UCSF Chimera and labeled on the bonds.

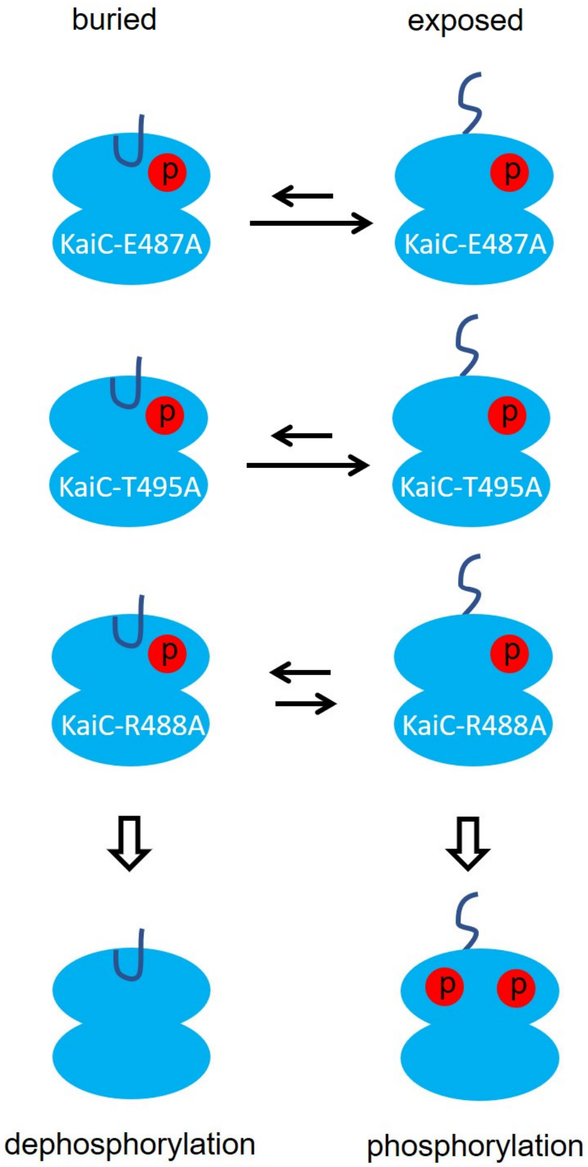

Figure S3. The dynamic equilibrium of KaiC mutants. The hypothetical equilibrium shift corresponds to the length of the arrow. The equilibrium shifts were predicted with the phosphorylation state of each KaiC mutant.

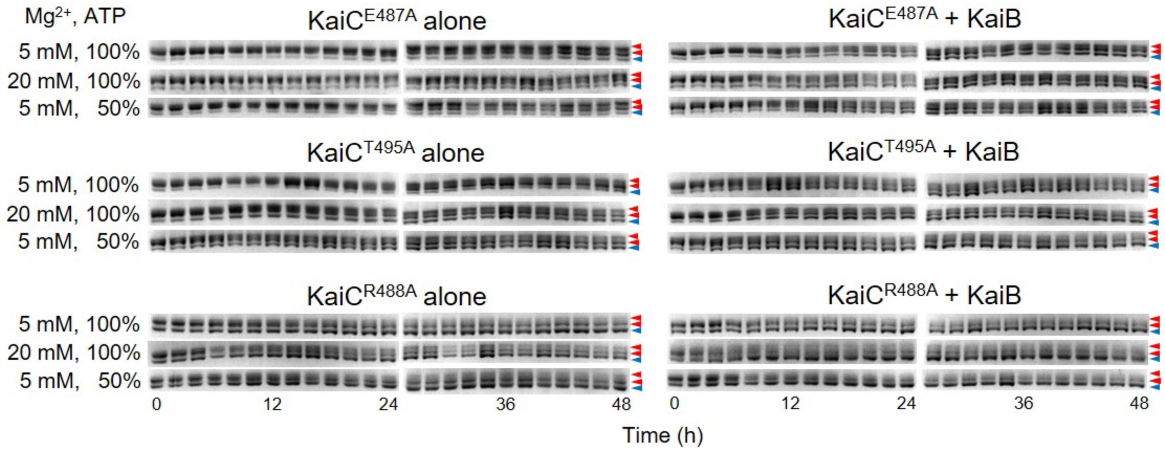

Figure S4. KaiC phosphorylation with various Mg^{2+} concentrations and ATP ratio. The phosphorylated (red triangle) and unphosphorylated (blue triangle) KaiC bands are separated by the 6.5% SDS-PAGE.

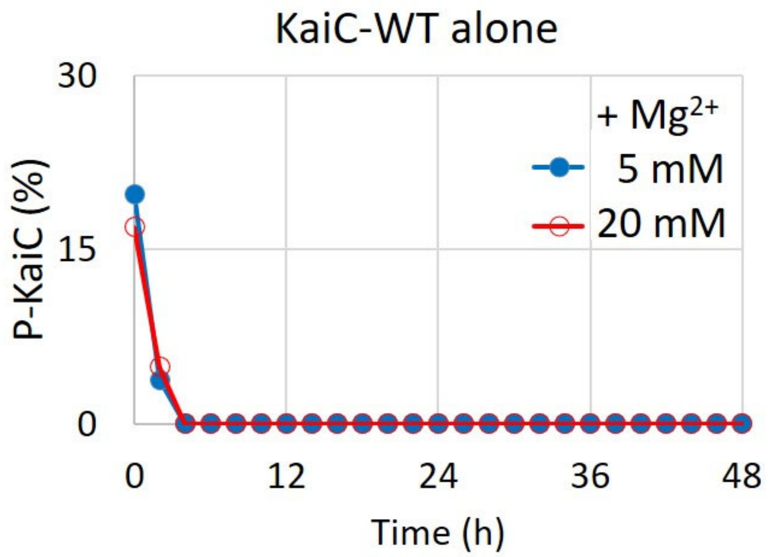

Figure S5. The dephosphorylation of KaiC in the presence of high Mg^{2+} concentration. Mg^{2+} concentrations are labeled on the legend. Circles are representing each data point.

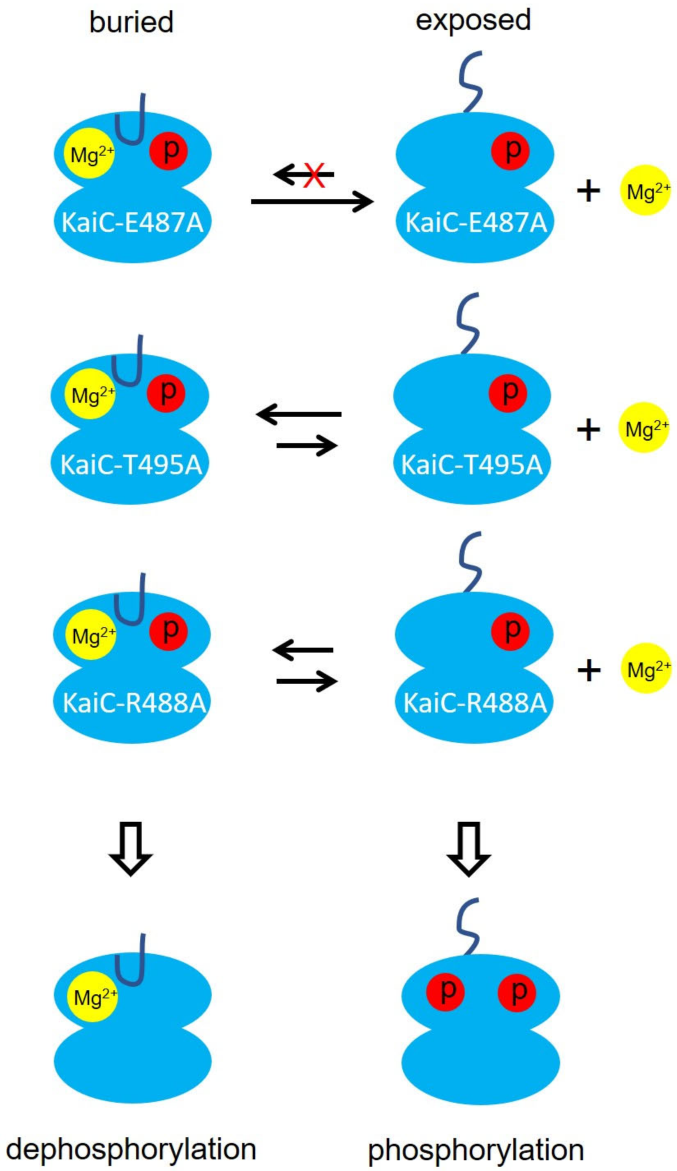

Figure S6. Dynamic equilibrium of KaiC mutants in the presence of high Mg^{2+} concentration. The hypothetical equilibrium shift corresponds to the length of the arrow. The equilibrium shifts were predicted with the phosphorylation state of each KaiC mutant.

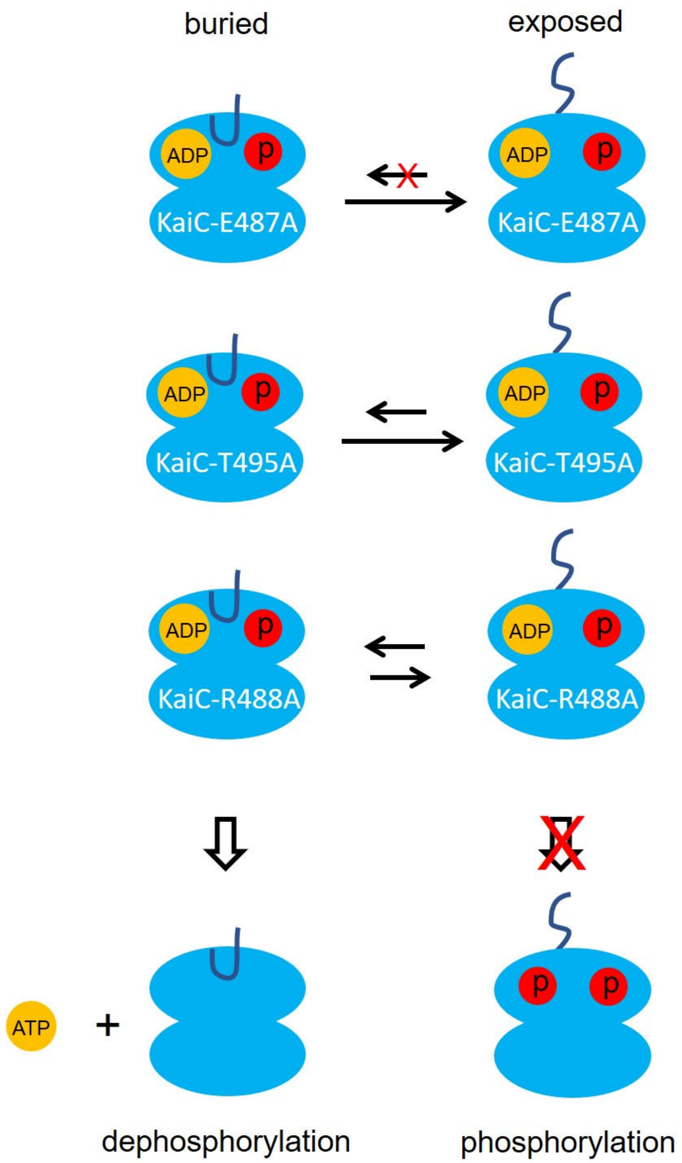

Figure S7. The dynamic equilibrium of KaiC mutants in the presence of high ADP concentration. ADP binds both conformations. The phosphorylation is inhibited when ADP is bound in the exposed conformation. The dephosphorylation is induced by producing ATP when ADP is bound in the buried conformation.

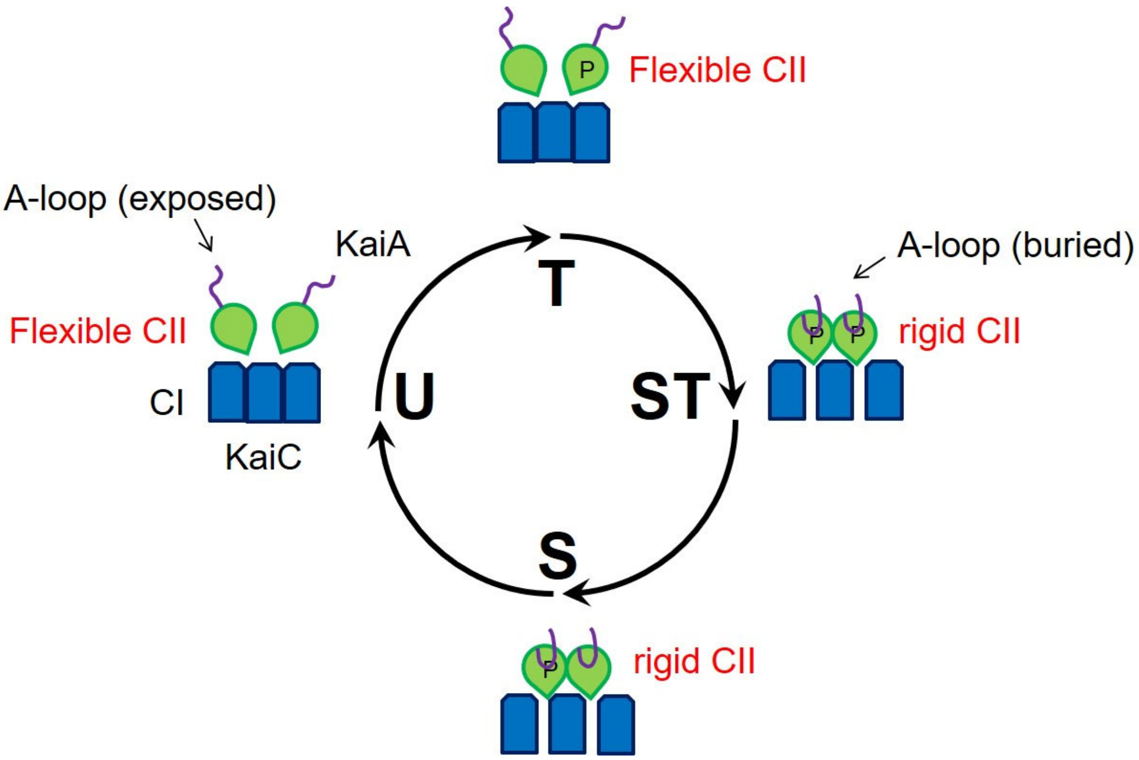

Figure S8. The flexibility change of the CII domain of KaiC throughout each phosphorylation state. The CII domains of KaiC are flexible in the unphosphorylated KaiC (U). Throughout the phosphorylation phase, the CII domains remain flexible (U → T → ST). The flexible CII domains allow the A-loop to be exposed. When KaiC is fully phosphorylated, the A-loop switches to the buried conformation in the rigid CII domains (ST) until it becomes fully dephosphorylated (ST → S → U). The major phosphorylation states (black letters) of KaiC and the direction (black arrows) are denoted as a cycle. The KaiC conformations corresponding to the phosphorylation states are also shown. To simplify, only 3 subunits of CI domain and 2 subunits of CII domain were drawn.

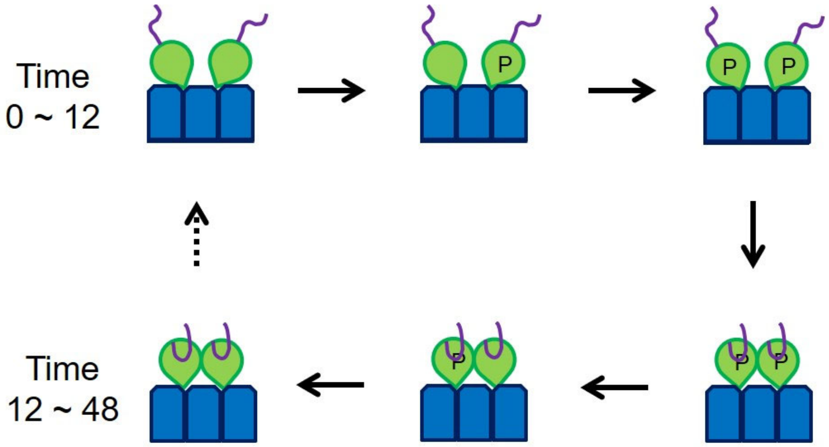

Figure S9. A model of the conformational changes during the KaiC phosphorylation and dephosphorylation. The conformational changes in the CII domain and A-loop induce spontaneous phosphorylation and dephosphorylation. Green balloons represent the CII domain and their position shows their flexibility. The dotted arrow is a hypothetical pathway that is not observed in the experiment.

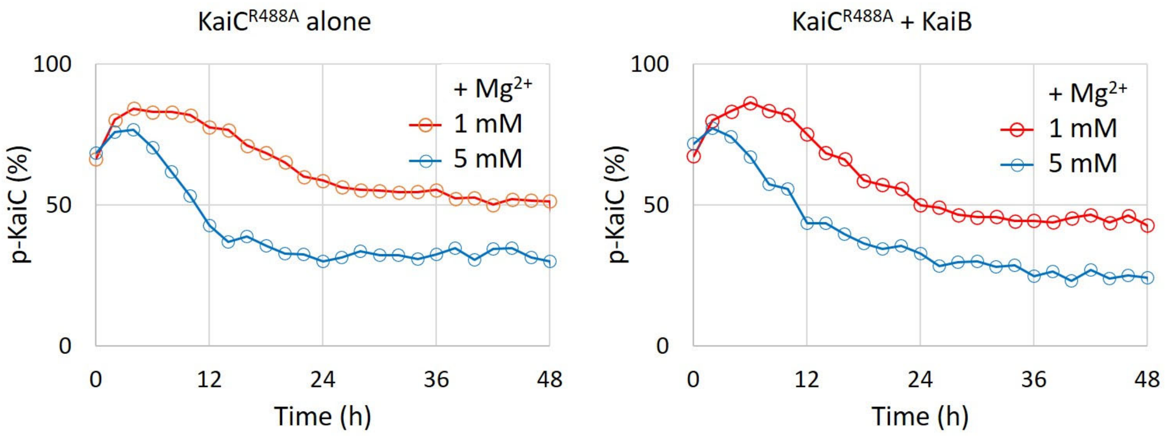

Figure S10. The spontaneous phosphorylation and dephosphorylation of KaiC^{R488A}. The phosphorylation state of KaiC^{R488A} was measured without KaiA (right) or KaiA and KaiB (left) every 2-hour for 2 days. The Mg^{2+} concentration of each reaction was labeled on the graph.

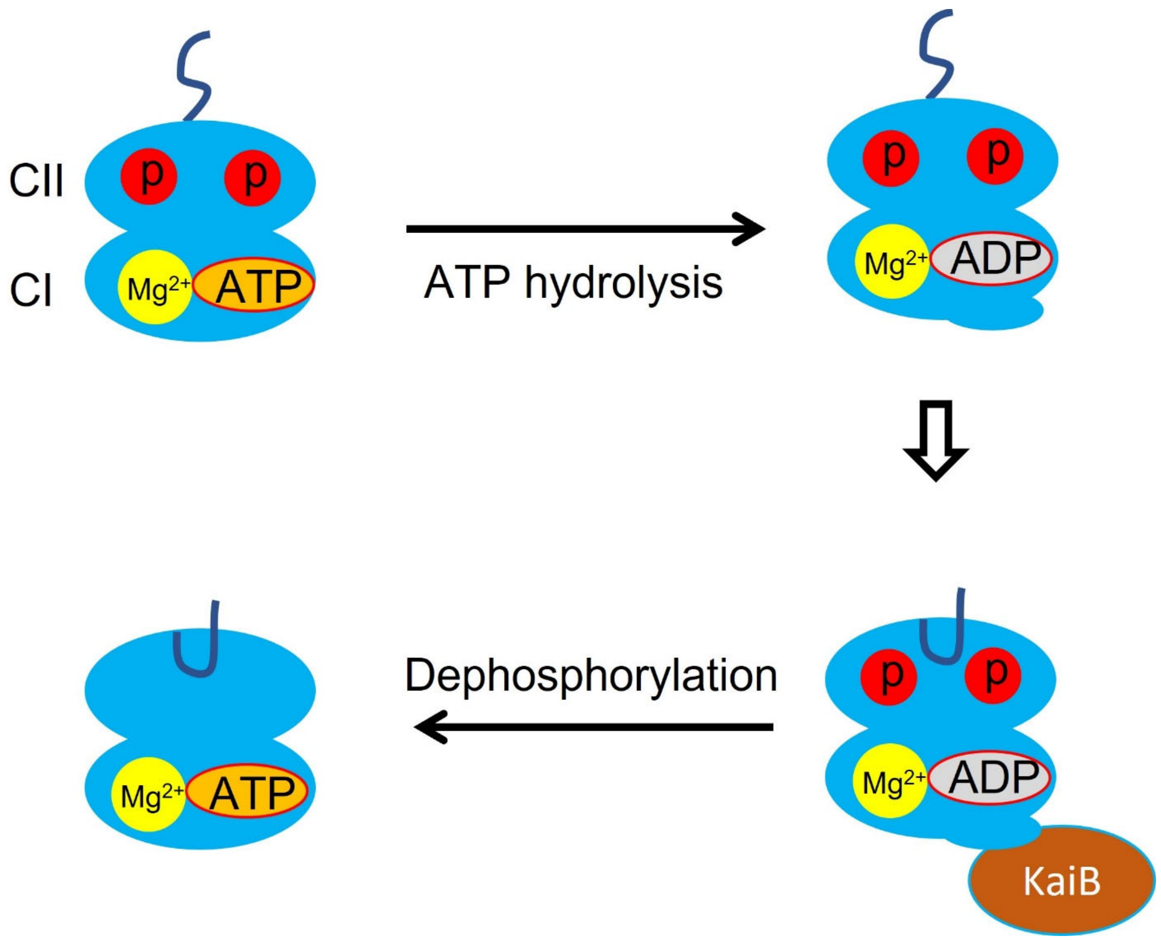

Figure S11. Mg^{2+} and ADP involvement on the ATP hydrolysis and KaiB binding on the CI domain of KaiC. Mg^{2+} and ATP may be elements necessary to change the CI conformation suitable for KaiB binding. Dephosphorylation on CII is reported to be facilitated by KaiB binding on CI [5,6]. When KaiC is fully phosphorylated, ATP bound to CI is hydrolyzed and generates ADP. The energy form the ATP hydrolysis change the conformation of CI to initiate KaiB binding. Also, presence of ADP on CI facilitates KaiB binding and dephosphorylation. When KaiB binds, KaiC switches its A-loop to the buried conformation and starts dephosphorylation.

References

1. Kim, P.; Kaszuba, A.; Jang, H.I.; Kim, Y.I. Purification of GST-Fused Cyanobacterial Central Oscillator Protein KaiC. *Applied Biochemistry and Microbiology* **2020**, *56*, 395–399, doi:10.1134/S0003683820040092.
2. Kim, Y.I.; Boyd, J.S.; Espinosa, J.; Golden, S.S. Detecting KaiC phosphorylation rhythms of the cyanobacterial circadian oscillator in vitro and in vivo. *Methods Enzymol.* **2015**, *551*, 153–173, doi:10.1016/bs.mie.2014.10.003.
3. Kim, Y.I.; Dong, G.; Carruthers, C.W., Jr.; Golden, S.S.; LiWang, A. The day/night switch in KaiC, a central oscillator component of the circadian clock of cyanobacteria. *Proc.Natl. Acad. Sci. USA* **2008**, *105*, 12825–12830, doi:10.1073/pnas.0800526105.
4. Pettersen, E.F.; Goddard, T.D.; Huang, C.C.; Couch, G.S.; Greenblatt, D.M.; Meng, E.C.; Ferrin, T.E. UCSF Chimera--a visualization system for exploratory research and analysis. *J Comput. Chem.* **2004**, *25*, 1605–1612, doi:10.1002/jcc.20084.
5. Phong, C.; Markson, J.S.; Wilhoite, C.M.; Rust, M.J. Robust and tunable circadian rhythms from differentially sensitive catalytic domains. *Proc. Natl. Acad. Sci. USA* **2013**, *110*, 1124–1129, doi:10.1073/pnas.1212113110.
6. Chang, Y.G.; Cohen, S.E.; Phong, C.; Myers, W.K.; Kim, Y.I.; Tseng, R.; Lin, J.; Zhang, L.; Boyd, J.S.; Lee, Y.; et al. Circadian rhythms. A protein fold switch joins the circadian oscillator to clock output in cyanobacteria. *Science* **2015**, *349*, 324–328, doi:10.1126/science.1260031.
